# On Local Anæsthesia from Cold, and Its Applicability to the Severer Operations

**Published:** 1862-08

**Authors:** James Arnott

**Affiliations:** York Street, Portman Square


### ON LOCAL ANÆSTHESIA FROM COLD, AND ITS APPLICABILITY TO THE SEVERER OPERATIONS.

By JAMES ARNOTT, M.D.

The recent occurrence, within a short period, of several deaths from chloroform, has again forcibly drawn the attention of the profession to the subject of anæsthesia in surgical operations. That chloroform and other general anæsthetics will by improved modes of administration, be deprived of their dangerous properties, is a hope now scarcely entertained, as evers effort to accomplish this, made during the last 14 years, has utterly failed; and the conviction has become general that it was unphilosophical, as being opposed to our former experience of analogous agencies, to expect that any means which so suddenly suspends both sensation and consciousness could be used without danger to life. The late recommendation of an American surgeon, that extreme or death-like drunkenness from alcohol should be substituted for the anæsthesia produced by ether and chloroform, is not on this account, likely to be well received.

Under these circumstances, it is natural to inquire whether the mode of producing the local insensibility by intense ar congealing cold, which has been practised and recommended by many surgeons as a perfectly safe and effectual expedient in minor operations, cannot be modified and extended to operations involving the incision of the deeper textures; and whether the suffering caused by these deep incisions, if it cannot be thus completely prevented, might not be so reduced or rendered tolerable as to make the administration of chloroform unnecessary.

The principal objection which was at first made to the production of anæsthesia by cold, that it was troublesome and tedious, it is now hardly worth while to notice. The trouble occasioned by mixing a little pulverized ice and salt, and applying the mixture by means of a bit of gauze for a minute or two to the skin (which is the ordinary mode of effecting congelation), has been found to be very little, and much less than what is required for the proper administration of chloroform. No previous minute examination of the heart and lungs is necessary for local anæsthesia; no assistant to watch the respiration and pulse, as well as to restrain the convulsive movements of the patient; no preparation of breathing and galvanic apparatus; for all of these, as we learn from the proceedings at coroners' inquests, are now expected by the public, and they all should be attended to and provided in order to lessen as much as possible the risk incurred.

Another objection to congelation was, that the reaction from it might interfere with the healing of the wound. But there is no reaction, in the proper sense of the term; instead of an increase there is a decrease of action as the result of congelation, and the antiphlogistic virtue proceeding from this effect is most valuable in preventing that excess of inflammation which so often prevents union by the first intention. This was strikingly shown by the different results of two operations performed on varicose veins in the Birmingham Hospital, one of which was done under anæsthesia from cold; and a list of nearly a hundred operations under congelation has been published by a London surgeon, in all but two of which the wound, when it was desired, healed by the first intention. Nor, so far as I know, has erysipelas ever succeeded an operation performed under this anæsthetic. It might, however, be otherwise if the congelation were by any means made to extend to the deeper tissues in the severer operations; for I have observed that when, in some of its remedial uses, it has been made to penetrate deeply, a very perceptible swelling has been the consequence, which might be adverse to union by the first intention, and, at all events, it would be necessary to bear this circumstance in the mind when dressing the wound.

Sir Charles Bell, in his Bridgewater Treatise on the Hand, has said that the skin is exquisitely sensitive in order that it may serve as a protector or monitor to the parts underneath, which, in their normal condition, are comparatively insensible; and that it is only when the skin is cut by the surgeon that acute pain is felt. If this statement be correct (and the opinion is not confined to Sir C. Bell), it would follow that any means capable of completely suspending the sensibility of the skin would deprive the deeper operations of their terrors. This cutaneous anæsthesia can certainly and on all occasions be effected by congelation, which, in its ordinary degree, not only thoroughly benumbs the skin, but the subcutaneous tissue also. And as the healing of the operation-wound is as certainly promoted by such moderate applications of this agent, it is cruel to withhold it in those amputations which are performed without chloroform, in consequence of the patient's extreme weakness or visceral disease rendering the danger from its use greater than usual.

It is undoubtedly a great defect of congelation, that its influence, in the ordinary mode of applying it, should extend to so small a depth. Whether the danger from chloroform ought not to be reckoned a much greater defect, in comparing the two agents, is a point on which opinions will probably continue to be divided. Those, however, who insist upon the necessity of perfect anæsthesia in operations, or at least of perfect forgetfulness by the patient of his having endured pain, may conjoin with congelation such a comparatively safe dose of chloroform as will be sufficient to render the deeper-seated tissues insensible.

Other modes of producing local anæsthesia have been proposed. In a treatise on Indigestion, published in 1847, I suggested the local application of aconitine and other narcotics, but their action has been found inadequate and uncertain; and electricity, at one time so much extolled for operations on the teeth, has proved a delusion. Local anæsthesia itself has suffered in character from the disappointment caused by the failure of these and similar expedients.

Having elsewhere described minutely the various methods of producing anæsthesia by cold, whether by the application of solids, liquids, or gases, already cooled to the requisite degree, or of bodies in a state of rapid transition from the solid to the fluid or aerial form, I shall at present limit myself to a very few remarks on this part of the subject. If sufficient pressure were combined with congelation, I have no doubt its influence would penetrate deeply enough to render amputation of the limbs perfectly painless. And this simultaneous pressure and congelation could easily be effected by enclosing a portion of the limb in a cylindrical case of gutta percha with membranous endings for tying, containing a powerful semi-fluid frigorfic mixture, and having rising from it a verticle tube, in order to contain conveniently the requisite hydraulic pressure. A flat, thin, metalic bottle, filled with such a mixture, or a solid piece of brass of the same shape already cooled to below zero of Fahr., would, by being passed slowly round the limb, constitute a convenient means of congealing the skin in amputation; and it is not improbable that the principle of the new mode of making ice by the sudden evaporation of ammonia may be found applicable to the construction of a simple apparatus for remedial congelation. If a dentist were willing to incur the necessary expense of time and money, he could, in the extraction of carious teeth, or in preparing the mouth for a set of artificial teeth, benumb the alveolar process very perfectly by covering the gums with a thin metalic case made to fit with the greatest accuracy by electrotype or otherwise, through a current of freezing brine could be passed. A current brings fresh particles of the cold body in quick succession, and the same advantage is obtained when a thin metalic vessel, containing a powerful freezing mixture, is both pressed and moved upon the part.

In the above observation I have made no allusion to the question whether the results of operations are affected by the chloroform taken to render them painless. When the operation is not severe, any increase of danger that might be produced by chloroform would not be appreciable; and certainly statistics show no increase of the mortality from these operations. It is very much otherwise, however, if the operation be in itself dangerous. Every reliable and extensive statistical report of such operations shows a great increase of mortality, all other circumstances being the same, since the introduction of chloroform. Nevertheless, many lives have been saved by etherization, for many patients would not have submitted to necessary operations without its use. As a remedy, also, of strangulated hernia, it has been highly and deservedly commended, but not in terms of higher eulogy than we find old authors bestowing on the similar virtues of tobacco. It is singular that both of these remedies of hernia should have the same defect. As respects tobacco, its fatal action in some few instances has led to its disuse. —*London Lancet.*

York Street, Portman Square, June, 1862.

